# Endoscopic therapy of stoma closure site strictures in ileal pouches is safe and effective

**DOI:** 10.1093/gastro/goae038

**Published:** 2024-05-16

**Authors:** Osama Jabi, Nan Lan, Akshay Pokala, Ravi P Kiran, Bo Shen

**Affiliations:** The Global Center for Integrated Colorectal Surgery and IBD Interventional Endoscopy, Columbia University Irving Medical Center/New York-Presbyterian Hospital, New York, NY, USA; The Global Center for Integrated Colorectal Surgery and IBD Interventional Endoscopy, Columbia University Irving Medical Center/New York-Presbyterian Hospital, New York, NY, USA; The Global Center for Integrated Colorectal Surgery and IBD Interventional Endoscopy, Columbia University Irving Medical Center/New York-Presbyterian Hospital, New York, NY, USA; The Global Center for Integrated Colorectal Surgery and IBD Interventional Endoscopy, Columbia University Irving Medical Center/New York-Presbyterian Hospital, New York, NY, USA; The Global Center for Integrated Colorectal Surgery and IBD Interventional Endoscopy, Columbia University Irving Medical Center/New York-Presbyterian Hospital, New York, NY, USA

**Keywords:** endoscopy, dilation, pouch, stricture, stoma closure, stricturotomy

## Abstract

**Background:**

Strictures are a common complication after ileal pouch surgery with the most common locations being at the anastomosis, pouch inlet, and stoma closure site. No previous literature has described endoscopic therapy of stoma site stricture. This study aimed to assess the safety and efficacy of endoscopic therapy in the treatment of stoma closure site strictures.

**Method:**

Patients diagnosed with stoma closure site strictures following ileal pouch surgery who underwent endoscopic treatment at the Center for Colorectal Diseases, Inflammatory Bowel Disease (IBD), and Ileal Pouch between 2018 and 2022 were analysed. Primary outcomes (technical success and surgery-free survival) were compared between endoscopic balloon dilation (EBD) and stricturotomy and/or strictureplasty.

**Results:**

A total of 30 consecutive eligible patients were analysed. Most patients were female (66.7%) and most patients were diagnosed with IBD (93.3%). Twenty patients (66.7%) had end-to-end anastomosis. A total of 52 procedures were performed, with EBD in 16 (30.8%) and stricturotomy and/or strictureplasty in 36 (69.2%). The mean stricture length was 1.7 ± 1.0 cm. Immediate technical success was achieved in 47 of 52 interventions (90.4%). During a mean follow-up of 12.7 ± 9.9 months, none of the patients underwent surgical intervention for the stricture. Fourteen (46.7%) required endoscopic re-intervention for their strictures with an interval between index and re-interventional pouchoscopy of 8.8 ± 6.3 months. Post-procedural complications were reported in 2 (6.7%) with bleeding and none with perforation. Upon follow-up, 20 (66.7%) patients reported improvement in their symptoms.

**Conclusion:**

EBD and endoscopic stricturotomy and/or strictureplasty are safe and effective in treating stoma closure site strictures in patients with ileal pouches, providing symptomatic relief in most patients as well as avoiding surgery.

## Introduction

Since its initial description back in 1978, restorative proctocolectomy with ileal pouch-anal anastomosis (IPAA) has become the surgical treatment of choice in patients with medically refractory ulcerative colitis, colitis-associated dysplasia, or familial adenomatous polyposis [1, 2]. IPAA has offered patients significant improvement in their quality of life and reduced their risk of disease complications, however, complications after IPAA can develop, such as pouchitis, cuffitis, Crohn’s disease (CD) of the pouch, fistulas, and strictures. Some of these can eventually lead to pouch failure with the need for repeated surgical intervention like revision or excision [3–6].

Strictures are a common complication after ileal pouch surgery, with a prevalence that can reach up to 38% [4, 7, 8]. The most common locations are at the pouch inlet, pouch outlet, anastomosis, and stoma closure site [9]. Management of pouch strictures can be challenging depending on the location and type of the stricture. The role of medical therapy in the treatment of pouch strictures is not clearly defined, although it may help in reducing the inflammation in patients with inflammatory strictures, the therapeutic effect on strictures of fibrotic nature is limited [10, 11].

The past decade has witnessed rapid advances in endoscopic therapy in the treatment of inflammatory bowel disease (IBD) or IBD surgery-associated complications, emerging as a safe and effective alternative to surgery for the treatment of pouch strictures. Various endoscopic approaches have been described, with the main two modalities for strictures being endoscopic balloon dilation (EBD) and endoscopic stricturotomy. Safety and efficacy of EBD in the treatment of pouch strictures have been reported with a significant improvement in patient’s quality of life and high pouch survival rates [9–12]. Endoscopic stricturotomy for the treatment of IBD-related strictures was first introduced in 2011 [9]. Since then this technique has been described in the treatment of anastomotic, inlet, and outlet strictures as well as nipple valve stenosis in patients with a continent ileostomy [13, 14]. Emerging as a more effective treatment than EBD and with a lower rate of perforation, yet, endoscopic stricturotomy has more risk of bleeding [11, 14]. Surgical intervention remains the last resort in patients with pouch strictures, it can be challenging due to the patient’s prior surgical history and the high risk for stricture recurrence and post-operative complications. However, the safety and efficacy of endoscopic therapy for stoma closure site strictures have not been described previously in the literature. This study aimed to assess the outcomes of interventional endoscopic therapy in the treatment of stoma closure site strictures.

## Patients and methods

### Data sources

All consecutive eligible patients with a diagnosis of stoma closure site strictures following ileal pouch surgery who underwent endoscopic treatment, with either EBD, endoscopic stricturotomy, and/or strictureplasty, between 2018 and 2022, were identified and evaluated through an approved pouch registry by Columbia University Irving Medical Center’s Institutional Review Board (NY, USA). Demographic, clinical, endoscopic, and patient outcomes were retrieved and carefully reviewed.

### Inclusion and exclusion criteria

The inclusion criteria for the study were patients having: (i) pelvic ileal pouches; (ii) strictures at the stoma closure site; and (iii) endoscopic treatment for the stricture, either with EBD or endoscopic stricturotomy and/or strictureplasty. The exclusion criteria were those with ileostomies.

### Demographic and clinical data

Demographics and clinical characteristics were retrieved as follows: age, gender, ethnicity, height, weight, body mass index, smoking history, past medical and surgical history in addition to family history. Disease and pouch characteristics were obtained from the charts and thoroughly assessed.

The diagnosis of strictures was based on a combined assessment of clinical presentation, imaging, and endoscopy. The characteristics of strictures were included, and the degree of stricture was classified based on whether the stricture was traversable with the scope. The length of the strictures was measured and reported during endoscopy, as well as having multiple strictures at different locations. Balloon sizes and knife types were also recorded.

### Techniques of endoscopic balloon dilation and stricturotomy

The endoscopic procedures were performed in outpatient or inpatient settings. Monitored anesthesia care was used. The patients prepared the bowel with polyethylene glycol-based oral solutions. Gastroscope and carbon dioxide insufflation were used for all procedures. The decision on EBD vs stricturotomy and the size of the balloon and depth of the electroincision was made at the discretion of the treating endoscopist (B.S.), based on nature (inflammatory vs fibrotic), orientation, and length of strictures. Antegrade or retrograde dilation with wire-guided or non-wire-guided through-the-scope controlled radial expansion balloons (Boston Scientific, Marlborough, MA, USA) was performed.

Endoscopic stricturotomy was performed in the same room and scope setting as EBD. Needle knife (Boston Scientific, Marlborough, MA, USA) or insulated-tip-2 knife was used for electroincision with an ERCP-I setting on ERBE (ERBE, Marietta, GA, USA). Patients with short (<1.5 cm) strictures with deep radial electroincision were treated with strictureplasty, ie stricturotomy plus placement of endoclips as spacers [15].

### Outcome measurements

The primary outcomes were technical success (defined as the passage of the scope through treated stricture) and surgery-free survival [15]. The secondary outcomes were endoscopic re-intervention-free survival and post-procedural complications such as perforation and bleeding. Follow-up time was recorded from the first endoscopic intervention to the last clinic appointment or telephone follow-up.

### Statistical analysis

Descriptive statistical analysis was performed for all variables. Categorical variables are reported as numbers (percentages). Quantitative variables are reported as mean ± standard deviation. The surgery-free survival and endoscopic re-intervention-free survival were evaluated using the Kaplan-Meier curves.

## Results

A total of 30 eligible patients who underwent ileal pouch surgery and were confirmed to have a stricture at their stoma closure site were included in this study (Table 1).

**Table 1.** Demographic and clinical features of patients (*n *=* *30) in this study

Characteristics	*n* (%)
Current age, years, mean ± SD	55.8 ± 12.0
Female	20 (66.7)
Race^a^	
White	21 (87.5)
Black/African American	0 (0)
Asian and other	3 (12.5)
Ethnicity^b^	
Hispanic or Latino	0 (0)
Not Hispanic and Latino	22 (100.0)
Body mass index, Kg/m^2^, mean ± SD	24.1 ± 4.7
Smoking history	
Never	21 (70.0)
Former	9 (30.0)
Current	0 (0)
Excessive alcohol	15 (50.0)
Significant comorbidities	22 (73.3)
Prior surgery	24 (80.0)
Family history	
Colon cancer	3 (10.0)
Inflammatory bowel disease	7 (23.3)
Prior chemotherapy	3 (10.0)
Underlying diagnosis	
Ulcerative colitis	24 (80.0)
Crohn’s disease	4 (13.3)
Indeterminate colitis	1 (3.3)
Familial adenomatous polyposis	1 (3.3)
Age at diagnosis, years, mean ± SD	25.3 ± 9.6
Indication for colectomy	
Refractory disease	14 (46.7)
Colitis associated neoplasia	6 (20.0)
Familial adenomatous polyposis	1 (3.3)
Colonic perforation	1 (3.3)
Perianal disease	11 (36.7)
Extraintestinal manifestations	12 (40.0)
J pouch	30 (100.0)
Staged pouch surgery	16 (53.3)
Surgical approach for pouch surgery^c^	
Open	6 (60.0)
Laparoscopic	4 (40.0)
Redo pouch	4 (13.3)
Stoma closure type	
End-to-end	20 (66.7)
Side-to-side	10 (33.3)
Symptoms	
Urgency	10 (33.3)
Nocturnal leak	3 (10.0)
Rectal pain	5 (16.7)
Perianal burning	3 (10.0)
Nausea/vomiting	7 (23.3)
Sense of incomplete evacuation	2 (6.7)
Abdominal pain	28 (93.3)
Straining	8 (26.7)
Rectal bleeding	6 (20.0)
Weight loss	6 (20.0)

a Due to missing data, the total number of patients was 24.

b Due to missing data, the total number of patients was 22.

c Due to missing data, the total number of patients was 10.

SD = standard deviation.

### Demographic and clinical features

Most patients were female (66.7%). The mean age at the time of the endoscopic procedure was 54.0 ± 10.7 years. Twenty-four patients (80.0%) had an underlying diagnosis of ulcerative colitis, 4 (13.3%) had CD, 1 (3.3%) had indeterminate colitis and 1 (3.3%) had familial adenomatous polyposis. For the stoma closure type, 20 (66.7%) patients had end-to-end anastomosis and 10 (33.3%) had side-to-side anastomosis. The most common reported symptom at presentation was abdominal pain in 28 (93.3%), followed by urgency in 10 (33.3%) and straining in 8 (26.7%). Before the endoscopic therapy, biologics use was reported in 13 (43.3%) patients (Table 1).

### Efficacy

**Figure 1.**
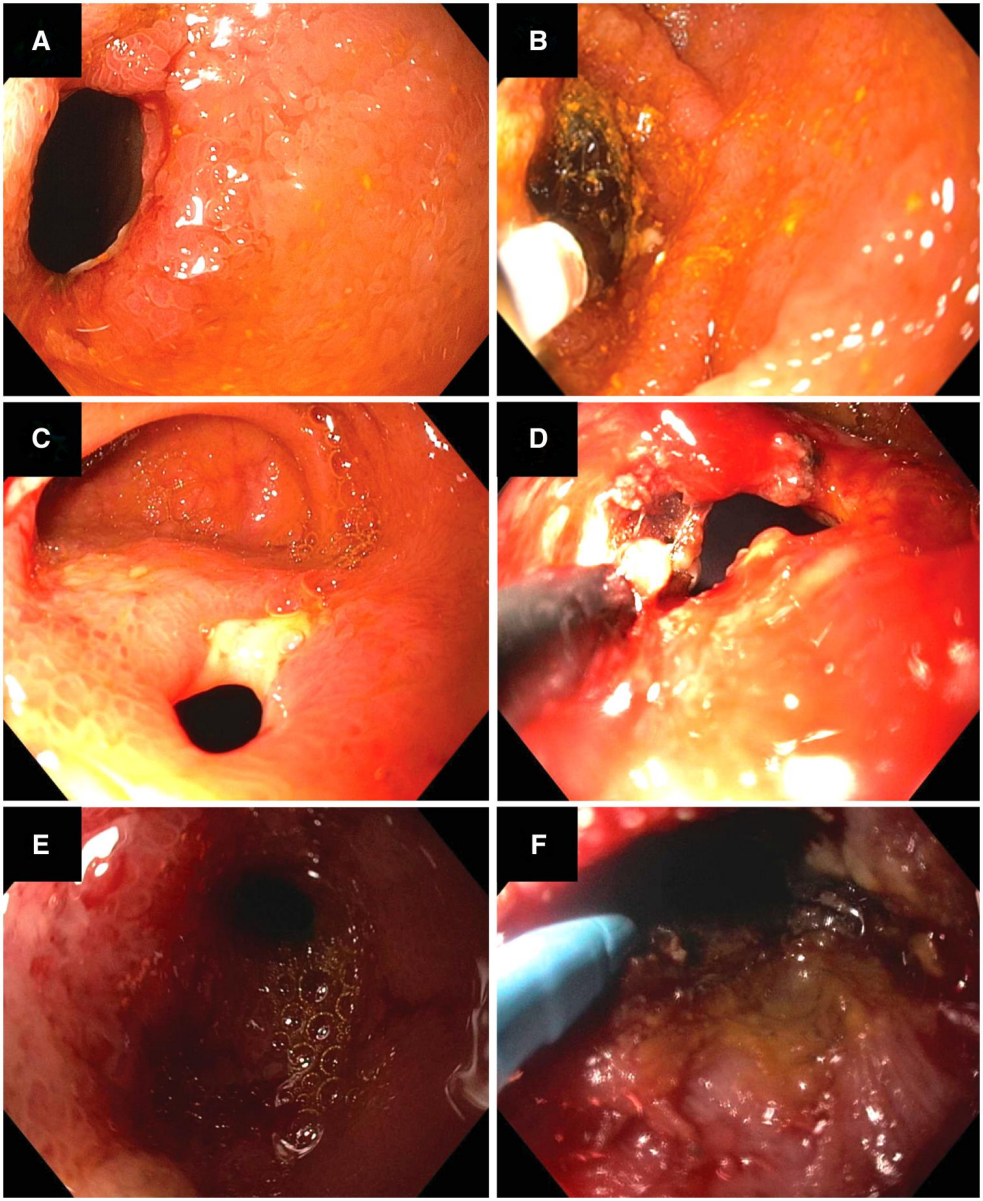
Images of clinical data before and after endoscopic treatment. (A) Before end-to-end endoscopic balloon dilation. (B) After end-to-end endoscopic balloon dilation. (C) Before side-to-side stricturotomy. (D) After side-to-side stricturotomy. (E) Before end-to-end stricturotomy. (F) After end-to-end stricturotomy.

A total of 52 procedures were performed for the cohort, with EBD in 16 (30.8%) and stricturotomy and/or strictureplasty in 36 (69.2%), with the insulated-tip-2 knife being the most commonly used (90.6%; Table 2 and Figure 1). The mean length of the stricture was 1.7 ± 1.0 cm. As for stricture degree, most patients had mild strictures (60.8%), 10 (19.6%) had moderate strictures, and 10 (19.6%) had severe or pinhole strictures. The use of biologics after the procedure was reported in 12 (40.0%) patients.

**Table 2.** Procedure details (*n *=* *52)

Characteristics	Value
Age at procedure, years, mean ± SD	54.0 ± 10.7
Pre-procedure medications	
Biologics	23 (44.2)
Corticosteroids	10 (19.2)
Immunomodulators	5 (9.6)
Antibiotics	20 (38.5)
Mesalamine	8 (15.4)
Small molecules	1 (1.9)
Stricture length, cm, mean ± SD	1.7 ± 1.0
Traversable prior to treatment	41 (78.8)
Stenosis degree^a^	
Mild	31 (60.8)
Moderate	10 (19.6)
Severe/pinhole	10 (19.6)
Ulcerated	17 (32.7)
Concurrent strictures at different locations	45 (86.5)
Intervention type	
Endoscopic balloon dilation	16 (30.8)
Stricturotomy	24 (46.2)
Strictureplasty	10 (19.2)
Stricturotomy and strictureplasty	2 (3.8)
Knife type^b^	
Insulation tipped	29 (90.6)
Needle knife	1 (3.1)
Insulation tipped and needle knife	2 (6.3)
Post-procedure medications	
Biologics	24 (46.2)
Corticosteroids	11 (21.2)
Immunomodulators	5 (9.6)
Antibiotics	21 (40.4)
Mesalamine	9 (17.3)
Small molecules	1 (1.9)

a Due to missing data, the total number was 51.

b Due to missing data, the total number was 32.

**Figure 2.**
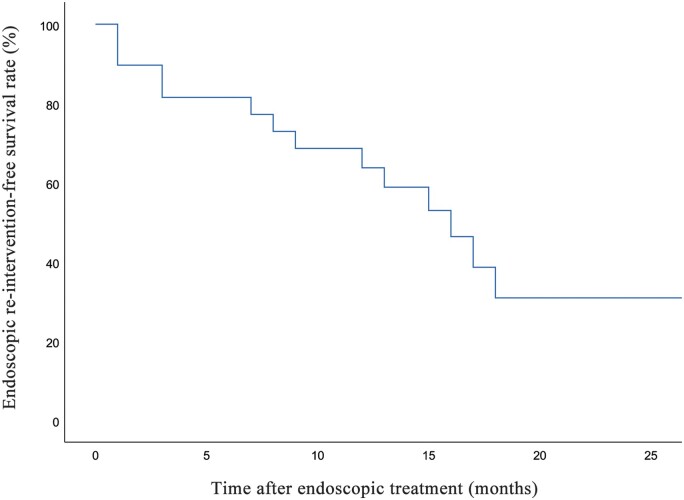
Kaplan-Meier curve for endoscopic re-intervention free survival.

During the follow-up period, symptomatic improvement was reported in 20 (66.7%) patients following the endoscopic intervention (Table 3). Immediate technical success was achieved in 47 of 52 interventions (90.4%). During a mean follow-up of 12.7 ± 9.9 months, none of the patients underwent surgical intervention for their stricture. Fourteen (46.7%) required endoscopic re-intervention for their strictures, with the interval between the index pouchoscopy and re-intervention pouchoscopy being 8.8 ± 6.3 months (Figure 2).

**Table 3.** Procedure outcomes of patients (*n *=* *30) in this study

Characteristics	Value
Immediately traversable^a^	47 (90.4)
Follow-up, months, mean ± SD	12.7 ± 9.9
Symptoms improved	20 (66.7)
Surgical intervention after therapy	0 (0.0)
Repeated endoscopic intervention	14 (46.7)
Index pouchoscopy to re-intervention pouchoscopy interval, months, mean ± SD	8.8 ± 6.3
Post procedural complications^b^	
None	22 (84.6)
Bleeding	2 (7.7)
Ileus	2 (7.7)
Emergency Department visit after procedure	4 (13.3)
Hospitalization after procedure	3 (10.0)

a *n *=* *52.

b Due to missing data, the total number of patients was 26.

Concurrent strictures at different locations such as the pouch inlet, anastomosis, or afferent limb were reported in 45 of 52 sessions (86.5%) and were treated in 43 (95.6%) including index and follow-up pouchoscopy.

### Comparison between endoscopic treatment modalities

**Figure 3.**
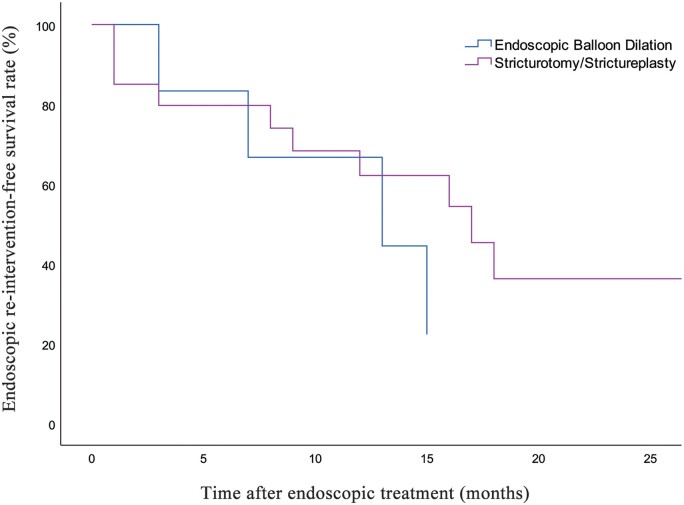
Kaplan-Meier curves for endoscopic re-intervention free survival in patients receiving endoscopic balloon dilation vs endoscopic stricturotomy/strictureplasty.

When evaluating the differences in outcome between treatment modalities, we found that out of the 20 patients with symptoms response, 7 (35.0%) had EBD as their index procedure and 13 (65.0%) had endoscopic stricturotomy and/or strictureplasty. The patients in the stricturotomy group and their counterparts in the EBD group had comparable endoscopic re-intervention-free survival (*P *=* *0.416) (Figure 3). Ten (71.4%) out of the 14 patients who required re-intervention for their strictures had stricturotomy and/or strictureplasty with the interval between the index pouchoscopy and re-intervention pouchoscopy being 8.6 ± 6.9 months and 4 (28.6%) had EBD with a mean interval of 9.5 ± 5.5 months. For patients who underwent re-intervention, the degree of stricture was measured during the index and first follow-up procedures, which showed that the stricturotomy group had 5 mild, 1 moderate, and 3 severe strictures at the index procedures compared with 7 mild, 2 moderate, and 1 severe at the first follow-up procedures. For the EBD group, patients had 2 mild and 2 moderate strictures at the index procedures compared with 3 mild and 1 moderate at the first follow-up procedures.

### Safety

Post-procedural complications were reported in 2 (6.7%) with bleeding, one of which required hospitalization, 2 (6.7%) with ileus, and none with perforation. A total of 4 patients (13.3%) visited the Emergency Department, 1 for bleeding, 1 for ileus, and 2 for abdominal pain (Table 3).

## Discussion

In this study, we evaluated 30 patients with ileal pouches and the impact of EBD and endoscopic stricturotomy and/or strictureplasty in treating patients with stoma closure site strictures. The majority of our patients had their stoma closed in an end-to-end fashion (66.7%). We showed that endoscopic intervention for strictures at this location can be safely performed in experienced hands, with an immediate technical success rate of 90.4%. None of our patients required surgical intervention for their pouches or the strictures during the follow-up period with 66.7% reporting significant improvement in their symptoms following the intervention. Multiple sessions of endoscopic therapy, however, were required in some patients (46.7%) with an interval of 8.8 ± 6.3 months between the first session and the re-intervention. Bleeding was documented in 2 patients who underwent endoscopic stricturotomy. We also found that the efficacy and safety of endoscopic dilation and endoscopic electroincision for stoma site strictures were comparable.

Pouch strictures remain a common complication after IPAA with contributing factors including surgery-associated ischemia, anastomotic tension, size of stapler used, the use of nonsteroidal anti-inflammatory drugs, CD of the pouch, and recurrent inflammation of the pouch [16, 17]. Strictures can be divided into inflammatory, fibrotic, or mixed [18, 19]. While inflammatory strictures might benefit from medical treatment given their role in reducing inflammation, fibrotic strictures are more challenging to treat with the limited effect of medical therapy, often necessitating the need for endoscopic or surgical intervention. In contrast to the latter with a high risk of stricture recurrence and postoperative complications, endoscopic therapy has emerged as a safe and effective alternative for surgery in the management of IBD strictures [20].

Endoscopic stricturotomy and EBD are the two most commonly used modalities for endoscopic therapy [9, 10]. The use of EBD has been widely studied in the treatment of IBD-related strictures. A meta-analysis of 24 studies with a total of 1,164 patients undergoing EBD for their strictures, where the majority of strictures were anastomotic, showed an immediate technical success rate of 89% and surgical intervention rate of 27% during a median follow-up of 15–70 months [21]. Another study looked at 150 patients with symptomatic pouch strictures at the pouch inlet and outlet, showed an immediate technical success rate of 97.8% and 80.3% reported some improvement in their symptoms following dilation [9].

Since its introduction, reports have shown endoscopic stricturotomy as a new and effective technique in treating IBD- and non-IBD-related strictures. A study on 85 patients with anastomotic strictures treated with endoscopic stricturotomy reported a 100% rate of immediate technical success and the need for surgical intervention in 15.3% [14]. On the other hand, a recent study evaluated the safety and efficacy of EBD vs endoscopic stricturotomy for the treatment of pouch inlet strictures in a total of 200 patients. Symptom improvement was recorded in 42.3% of patients treated with endoscopic stricturotomy and 13.2% of patients treated with EBD. There were three events of bleeding with endoscopic stricturotomy and three events of perforation with EBD [11].

Thorough evaluation of the ileal pouch has not been standardized until the recent publication of a consensus guideline by the International Ileal Pouch Consortium [22]. In the guideline, a quality pouchoscopy should include an evaluation of the stoma closure site, prepouch ileum, inlet, pouch body, cuff, and anal transitional zone. The distance between the pouch inlet and the stoma closure site usually spans 15 cm to 25 cm from the pouch inlet.

There is a lack of published studies on the management of strictures of stoma closure site. Our study showed an immediate technical success rate of 90.4%, which is similar to that reported in the literature on endoscopic therapy in IBD strictures. Our study also showed a 66.7% rate of symptom improvement as well as a low rate of adverse events with two of the patients receiving endoscopic stricturotomy experiencing episodes of bleeding, similar to previous reports in the literature of post-procedure bleeding favoring endoscopic stricturotomy over EBD. Despite the fact that our study had a small cohort, our surgical intervention rate of 0% is lower than what’s reported in previous studies and none of our patients treated with EBD experienced an event of perforation.

The length of stricture might affect the response to endoscopic therapy. A recent study showed that a stricture length of < 4 cm was associated with a lower risk of surgical intervention [21]. In our study, the mean length of the stricture was 1.7 ± 1.0 cm. We encountered one patient who had a stricture of ≥ 4 cm requiring the highest number of endoscopic therapy sessions with failure of technical success.

Intervention endoscopy plays an important role in the management of IBD strictures [23]. While the impact of interventional endoscopic therapy has been studied on strictures in different locations such as pouch inlet, outlet, or anastomosis [24], the role of this innovative therapy has not been yet studied on stricture at the stoma closure site. The findings of our study have several clinical implications. Our study shows that EBD and endoscopic stricturotomy and/or strictureplasty are safe and effective in treating stoma closure site strictures in patients with ileal pouches, providing symptomatic relief in most patients with findings similar to what’s reported in previous studies on strictures in different locations. Our findings may be extrapolated to the treatment of strictures of stoma closure sites in non-pouch patients, such as those with CD and temporary diverting ileostomy. As symptomatic strictures at the stoma closure site are traditionally treated with surgery [25], our study provides evidence that endoscopic therapy can successfully treat strictures at this location and may help patients avoid surgery or prolong the need for one. For patients with pouch strictures refractory to endoscopic therapy, the diagnosis of CD of the pouch should be considered. Patients with CD strictures require concurrent medical therapy [26, 27].

This study has limitations. It’s a single-center case series with a small sample size and a short follow-up period. Larger prospective or case-controlled studies with multicenter collaboration and direct comparison between endoscopic stricturotomy and EBD are needed to better understand the impact of interventional endoscopy on strictures at the stoma closure site and if surgical techniques employed in stoma reversal has any effect on developing strictures at the location.

## Conclusion

Both EBD and endoscopic stricturotomy and/or strictureplasty appear to be safe and effective in treating patients with strictures at the stoma closure site following restorative proctocolectomy and IPAA.

## Authors’ Contributions

B.S. and R.K. conceived and designed the study; O.J., N.L., and A.P. acquired the data; O.J., N.L., and B.S. analysed and interpreted the data; O.J., N.L. A.P., and B.S. drafted the article. All authors have read and approved the final version of the manuscript.
